# Bis{2-[(4-bromo­phen­yl)imino­meth­yl]pyridine-κ^2^ *N*,*N*′}copper(I) tetra­phenyl­borate

**DOI:** 10.1107/S1600536808020679

**Published:** 2008-07-09

**Authors:** Ali Mahmoudi, Maryam Hajikazemi, Mehdi Khalaj, Saeed Dehghanpour

**Affiliations:** a Department of Chemistry, Islamic Azad University, Karaj Branch, Karaj, Iran; b Department of Chemistry, Alzahra University, Vanak, PO Box 1993891176, Tehran, Iran

## Abstract

In the crystal structure of the title compound, [Cu(C_12_H_9_BrN_2_)_2_](C_24_H_20_B), the copper(I) cation is coordinated by four N atoms of two crystallographically independent 2-[(4-bromo­phen­yl)imino­meth­yl]pyridine ligands within a distorted tetra­hedron.

## Related literature

For applications of imino­pyridine complexes, see: Armaroli (2001 ▶); Sakaki *et al.* (2002 ▶). For related structures, see Dehghanpour & Mahmoudi (2007 ▶); Dehghanpour *et al.* (2007 ▶).
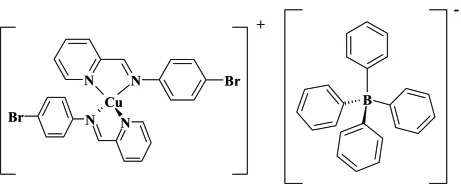


## Experimental

### 

#### Crystal data

[Cu(C_12_H_9_BrN_2_)_2_](C_24_H_20_B)
*M* *_r_* = 904.99
Triclinic, 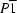

*a* = 11.7198 (10) Å
*b* = 13.1527 (11) Å
*c* = 14.4735 (12) Å
α = 80.5034 (9)°
β = 69.3835 (8)°
γ = 89.5465 (9)°
*V* = 2056.5 (3) Å^3^
*Z* = 2
Mo *K*α radiation
μ = 2.51 mm^−1^
*T* = 193 (2) K
0.67 × 0.37 × 0.34 mm

#### Data collection

Bruker SMART 1000 CCD area-detector/PLATFORM diffractometer
Absorption correction: integration (*SHELXTL*; Sheldrick, 2008 ▶) *T*_min_ = 0.339, *T*_max_ = 0.423
17095 measured reflections
9230 independent reflections
7124 reflections with *I* > 2σ(*I*)
*R*_int_ = 0.022

#### Refinement

*R*[*F* ^2^ > 2σ(*F* ^2^)] = 0.033
*wR*(*F* ^2^) = 0.079
*S* = 1.03
9230 reflections
505 parameters
H-atom parameters constrained
Δρ_max_ = 0.70 e Å^−3^
Δρ_min_ = −0.39 e Å^−3^

### 

Data collection: *SMART* (Bruker, 1997 ▶); cell refinement: *SAINT* (Bruker, 1997 ▶); data reduction: *SAINT*; program(s) used to solve structure: *DIRDIF99* (Beurskens *et al.*, 1999 ▶); program(s) used to refine structure: *SHELXL97* (Sheldrick, 2008 ▶); molecular graphics: *SHELXTL* (Sheldrick, 2008 ▶); software used to prepare material for publication: *SHELXTL*.

## Supplementary Material

Crystal structure: contains datablocks global, I. DOI: 10.1107/S1600536808020679/nc2107sup1.cif

Structure factors: contains datablocks I. DOI: 10.1107/S1600536808020679/nc2107Isup2.hkl

Additional supplementary materials:  crystallographic information; 3D view; checkCIF report

## Figures and Tables

**Table:** 

Cu—N1	2.0158 (17)
Cu—N4	2.0237 (16)
Cu—N3	2.0278 (17)
Cu—N2	2.0331 (16)

**Table:** 

N1—Cu—N4	135.35 (7)
N1—Cu—N3	120.47 (7)
N4—Cu—N3	81.51 (7)
N1—Cu—N2	82.60 (7)
N4—Cu—N2	122.20 (7)
N3—Cu—N2	119.62 (7)

**Fig. 1.**
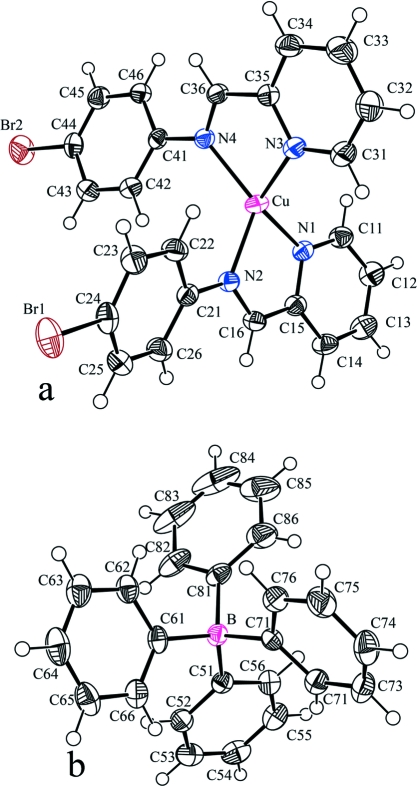
Crystal structure of the cation (a) and the anion (b) with labeling and thermal ellipsoids drawn at 50% the probability level. Hydrogen atoms are shown as spheres of arbitrary radius.

## supplementary crystallographic information

### Comment

Much interest has recently been focused on the rational design and construction of novel copper(I) complexes because of their reversible electrochemical behavior, light absorption in the visible spectral region; characteristic structural flexibility, long-lived electronically excited states, intense luminescence and ease of preparation (Armaroli, 2001; Sakaki *et al.*, 2002). In continuation of our interests on this topic (Dehghanpour *et al.*, 2007; Dehghanpour & Mahmoudi, 2007), we report herein the X-ray crystal structure of the copper(I) complex of the Schiff base ligand of (4-bromo-phenyl)-pyridin-2-ylmethylene-amine.

The structure of (I) consists of discrete [(C_12_H_9_BrN_2_)_2_Cu]^+^ cations and [BPh_4_]^-^ anions (Fig. 1). The copper(I) cation centre has a tetrahedral coordination which shows signficant distortion, mainly due to the presence of the five-membered chelate ring (Table 1). The endocyclic N1—Cu—N2 angle is much smaller than the ideal tetrahedral angle of 109.5^°^, whereas the opposite N1—Cu—N4 angle is much wider than the ideal tetrahedral angle.

### Experimental

To a solution of (4-bromo-phenyl)-pyridin-2-ylmethylene-amine (37.8 mg, 0.1 mmol) in 20 ml acetonitrile copper tetraphenylborate (28.9 mg, 0.1 mmol)was added . The mixture was heated to dissolve the reactants, filtered off and the solvent was removed under vacuum to about 5 ml. The diffusion of diethyl ether vapor into the solution leads to light-yellow crystals. The crystals were collected and washed with diethylether. yield 83%. Calc. for C_48_H_38_BBr_2_CuN_4_: C 63.70, H 4.23, N 6.19%; found: C 63.73, H 4.21, N 6.17%.

### Refinement

All hydrogen atoms were placed in geometrically calculated positions and refined isotropic using a riding model with *U*_iso_(H) equal to 1.2Ueq(C).

### Crystal data

**Table:**

[Cu(C_12_H_9_BrN_2_)_2_](C_24_H_20_B)	*Z* = 2
*M**_r_* = 904.99	*F*_000_ = 916
Triclinic, *P*1	*D*_x_ = 1.461 Mg m^−^^3^
Hall symbol: -P 1	Mo *K*α radiation λ = 0.71073 Å
*a* = 11.7198 (10) Å	Cell parameters from 7821 reflections
*b* = 13.1527 (11) Å	θ = 2.4–27.3º
*c* = 14.4735 (12) Å	µ = 2.51 mm^−^^1^
α = 80.5034 (9)º	*T* = 193 (2) K
β = 69.3835 (8)º	Prism, brown
γ = 89.5465 (9)º	0.67 × 0.37 × 0.34 mm
*V* = 2056.5 (3) Å^3^	

### Data collection

**Table:**

Bruker SMART 1000 CCD area-detector/PLATFORM diffractometer	9230 independent reflections
Radiation source: fine-focus sealed tube	7124 reflections with *I* > 2σ(*I*)
Monochromator: graphite	*R*_int_ = 0.022
Detector resolution: 8.192 pixels mm^-1^	θ_max_ = 27.5º
*T* = 193(2) K	θ_min_ = 1.6º
ω scans	*h* = −15→15
Absorption correction: integration(SHELXTL; Sheldrick, 2008)	*k* = −17→17
*T*_min_ = 0.339, *T*_max_ = 0.423	*l* = −18→18
17095 measured reflections	

### Refinement

**Table:**

Refinement on *F*^2^	Secondary atom site location: difference Fourier map
Least-squares matrix: full	Hydrogen site location: inferred from neighbouring sites
*R*[*F*^2^ > 2σ(*F*^2^)] = 0.033	H-atom parameters constrained
*wR*(*F*^2^) = 0.079	*w* = 1/[σ^2^(*F*_o_^2^) + (0.0381*P*)^2^ + 0.4988*P*] where *P* = (*F*_o_^2^ + 2*F*_c_^2^)/3
*S* = 1.03	(Δ/σ)_max_ = 0.001
9230 reflections	Δρ_max_ = 0.70 e Å^−^^3^
505 parameters	Δρ_min_ = −0.39 e Å^−^^3^
Primary atom site location: structure-invariant direct methods	Extinction correction: none

### Special details

**Table:**

Geometry. All e.s.d.'s (except the e.s.d. in the dihedral angle between two l.s. planes) are estimated using the full covariance matrix. The cell e.s.d.'s are taken into account individually in the estimation of e.s.d.'s in distances, angles and torsion angles; correlations between e.s.d.'s in cell parameters are only used when they are defined by crystal symmetry. An approximate (isotropic) treatment of cell e.s.d.'s is used for estimating e.s.d.'s involving l.s. planes.
Refinement. Refinement of *F*^2^ against ALL reflections. The weighted *R*-factor *wR* and goodness of fit *S* are based on *F*^2^, conventional *R*-factors *R* are based on *F*, with *F* set to zero for negative *F*^2^. The threshold expression of *F*^2^ > σ(*F*^2^) is used only for calculating *R*-factors(gt) *etc*. and is not relevant to the choice of reflections for refinement. *R*-factors based on *F*^2^ are statistically about twice as large as those based on *F*, and *R*- factors based on ALL data will be even larger.

### Fractional atomic coordinates and isotropic or equivalent isotropic displacement parameters (Å^2^)

**Table:**

	*x*	*y*	*z*	*U*_iso_*/*U*_eq_	
Br1	0.49211 (2)	−0.06504 (2)	0.16116 (2)	0.04820 (8)	
Br2	0.47991 (2)	0.72688 (2)	−0.05911 (2)	0.05109 (8)	
Cu	0.00222 (2)	0.30630 (2)	0.192415 (18)	0.03041 (7)	
N1	−0.10338 (15)	0.35066 (13)	0.32107 (12)	0.0263 (4)	
N2	0.09083 (15)	0.23492 (13)	0.28111 (12)	0.0250 (4)	
N3	−0.07144 (16)	0.22149 (14)	0.12033 (13)	0.0295 (4)	
N4	0.09239 (14)	0.37880 (13)	0.05029 (12)	0.0244 (3)	
C11	−0.2061 (2)	0.40192 (17)	0.34380 (16)	0.0332 (5)	
H11	−0.2324	0.4319	0.2908	0.040*	
C12	−0.2761 (2)	0.41345 (18)	0.44094 (18)	0.0379 (5)	
H12	−0.3497	0.4488	0.4540	0.045*	
C13	−0.2376 (2)	0.37322 (18)	0.51748 (17)	0.0397 (5)	
H13	−0.2835	0.3810	0.5844	0.048*	
C14	−0.1315 (2)	0.32138 (18)	0.49650 (16)	0.0351 (5)	
H14	−0.1024	0.2938	0.5485	0.042*	
C15	−0.06756 (18)	0.31009 (16)	0.39809 (15)	0.0272 (4)	
C16	0.03963 (19)	0.24799 (16)	0.37211 (15)	0.0280 (4)	
H16	0.0708	0.2178	0.4220	0.034*	
C21	0.19111 (18)	0.17015 (16)	0.25376 (15)	0.0261 (4)	
C22	0.2140 (2)	0.13170 (18)	0.16535 (16)	0.0331 (5)	
H22	0.1664	0.1520	0.1249	0.040*	
C23	0.3057 (2)	0.06406 (18)	0.13577 (18)	0.0366 (5)	
H23	0.3221	0.0387	0.0748	0.044*	
C24	0.37296 (19)	0.03402 (17)	0.19602 (17)	0.0331 (5)	
C25	0.3544 (2)	0.07447 (19)	0.28212 (17)	0.0369 (5)	
H25	0.4034	0.0550	0.3216	0.044*	
C26	0.2645 (2)	0.14320 (18)	0.31047 (17)	0.0341 (5)	
H26	0.2526	0.1723	0.3689	0.041*	
C31	−0.1550 (2)	0.14279 (18)	0.15765 (18)	0.0386 (5)	
H31	−0.1820	0.1162	0.2274	0.046*	
C32	−0.2038 (2)	0.0985 (2)	0.0986 (2)	0.0489 (7)	
H32	−0.2641	0.0432	0.1276	0.059*	
C33	−0.1640 (2)	0.1356 (2)	−0.0027 (2)	0.0497 (7)	
H33	−0.1970	0.1067	−0.0444	0.060*	
C34	−0.0754 (2)	0.21534 (18)	−0.04318 (17)	0.0374 (5)	
H34	−0.0452	0.2411	−0.1132	0.045*	
C35	−0.03165 (18)	0.25667 (16)	0.02032 (15)	0.0274 (4)	
C36	0.06125 (18)	0.34185 (16)	−0.01476 (15)	0.0268 (4)	
H36	0.0978	0.3693	−0.0842	0.032*	
C41	0.18453 (17)	0.46002 (15)	0.01951 (14)	0.0242 (4)	
C42	0.25621 (19)	0.46230 (17)	0.07779 (16)	0.0308 (5)	
H42	0.2433	0.4101	0.1349	0.037*	
C43	0.3465 (2)	0.53988 (18)	0.05357 (17)	0.0347 (5)	
H43	0.3964	0.5406	0.0929	0.042*	
C44	0.36266 (19)	0.61608 (17)	−0.02864 (17)	0.0320 (5)	
C45	0.2927 (2)	0.61523 (17)	−0.08761 (17)	0.0337 (5)	
H45	0.3052	0.6682	−0.1440	0.040*	
C46	0.20372 (19)	0.53644 (16)	−0.06401 (15)	0.0289 (4)	
H46	0.1560	0.5347	−0.1049	0.035*	
C51	0.06498 (18)	0.24478 (16)	0.67108 (15)	0.0272 (4)	
C52	−0.0367 (2)	0.17432 (18)	0.70596 (15)	0.0320 (5)	
H52	−0.0238	0.1036	0.7016	0.038*	
C53	−0.1559 (2)	0.2044 (2)	0.74676 (17)	0.0400 (6)	
H53	−0.2223	0.1545	0.7685	0.048*	
C54	−0.1784 (2)	0.3061 (2)	0.75572 (17)	0.0421 (6)	
H54	−0.2596	0.3267	0.7829	0.050*	
C55	−0.0807 (2)	0.37743 (19)	0.72457 (17)	0.0400 (5)	
H55	−0.0944	0.4475	0.7315	0.048*	
C56	0.0373 (2)	0.34674 (17)	0.68315 (16)	0.0325 (5)	
H56	0.1028	0.3974	0.6619	0.039*	
C61	0.23163 (19)	0.09653 (16)	0.60873 (15)	0.0286 (4)	
C62	0.3289 (2)	0.04312 (17)	0.62508 (16)	0.0328 (5)	
H62	0.3750	0.0735	0.6569	0.039*	
C63	0.3606 (2)	−0.05214 (18)	0.59683 (18)	0.0407 (6)	
H63	0.4281	−0.0848	0.6084	0.049*	
C64	0.2946 (2)	−0.09949 (18)	0.55202 (19)	0.0448 (6)	
H64	0.3161	−0.1647	0.5327	0.054*	
C65	0.1971 (2)	−0.05103 (18)	0.53558 (18)	0.0427 (6)	
H65	0.1504	−0.0831	0.5053	0.051*	
C66	0.1670 (2)	0.04504 (17)	0.56331 (16)	0.0352 (5)	
H66	0.0996	0.0771	0.5509	0.042*	
C71	0.28089 (18)	0.28595 (15)	0.51315 (15)	0.0264 (4)	
C72	0.2281 (2)	0.35601 (16)	0.45882 (16)	0.0314 (5)	
H72	0.1451	0.3707	0.4899	0.038*	
C73	0.2919 (2)	0.40563 (18)	0.36052 (17)	0.0392 (5)	
H73	0.2518	0.4523	0.3262	0.047*	
C74	0.4128 (2)	0.38701 (19)	0.31335 (18)	0.0437 (6)	
H74	0.4563	0.4200	0.2465	0.052*	
C75	0.4692 (2)	0.3197 (2)	0.36488 (19)	0.0450 (6)	
H75	0.5527	0.3065	0.3336	0.054*	
C76	0.4051 (2)	0.27106 (18)	0.46188 (17)	0.0368 (5)	
H76	0.4467	0.2255	0.4956	0.044*	
C81	0.2680 (2)	0.24384 (17)	0.70707 (17)	0.0323 (5)	
C82	0.2269 (2)	0.1840 (2)	0.80372 (18)	0.0464 (6)	
H82	0.1689	0.1281	0.8186	0.056*	
C83	0.2680 (3)	0.2037 (3)	0.8778 (2)	0.0700 (11)	
H83	0.2381	0.1613	0.9421	0.084*	
C84	0.3510 (4)	0.2833 (3)	0.8598 (3)	0.0828 (13)	
H84	0.3788	0.2967	0.9111	0.099*	
C85	0.3942 (3)	0.3443 (3)	0.7657 (3)	0.0751 (11)	
H85	0.4520	0.4001	0.7522	0.090*	
C86	0.3530 (3)	0.3240 (2)	0.6902 (2)	0.0524 (7)	
H86	0.3842	0.3663	0.6258	0.063*	
B	0.2094 (2)	0.21697 (18)	0.62532 (17)	0.0264 (5)	

### Atomic displacement parameters (Å^2^)

**Table:**

	*U*^11^	*U*^22^	*U*^33^	*U*^12^	*U*^13^	*U*^23^
Br1	0.03103 (13)	0.04432 (15)	0.07066 (19)	0.01163 (10)	−0.01537 (12)	−0.02038 (13)
Br2	0.04489 (15)	0.04317 (15)	0.06251 (18)	−0.01618 (11)	−0.01933 (13)	−0.00032 (13)
Cu	0.03340 (15)	0.03807 (16)	0.01943 (13)	0.00175 (11)	−0.00992 (11)	−0.00288 (11)
N1	0.0289 (9)	0.0250 (9)	0.0233 (9)	−0.0006 (7)	−0.0080 (7)	−0.0022 (7)
N2	0.0259 (8)	0.0265 (9)	0.0228 (9)	0.0005 (7)	−0.0092 (7)	−0.0037 (7)
N3	0.0306 (9)	0.0318 (10)	0.0243 (9)	−0.0026 (8)	−0.0091 (8)	−0.0009 (7)
N4	0.0240 (8)	0.0255 (9)	0.0226 (9)	0.0031 (7)	−0.0075 (7)	−0.0027 (7)
C11	0.0364 (12)	0.0323 (12)	0.0311 (12)	0.0058 (9)	−0.0130 (10)	−0.0035 (9)
C12	0.0378 (12)	0.0357 (13)	0.0383 (13)	0.0114 (10)	−0.0099 (11)	−0.0096 (10)
C13	0.0466 (14)	0.0406 (13)	0.0293 (12)	0.0072 (11)	−0.0067 (11)	−0.0138 (10)
C14	0.0422 (13)	0.0402 (13)	0.0243 (11)	0.0055 (10)	−0.0121 (10)	−0.0090 (10)
C15	0.0302 (11)	0.0273 (11)	0.0251 (10)	−0.0011 (8)	−0.0103 (9)	−0.0063 (8)
C16	0.0303 (11)	0.0308 (11)	0.0255 (11)	0.0027 (9)	−0.0134 (9)	−0.0048 (9)
C21	0.0263 (10)	0.0259 (10)	0.0240 (10)	−0.0011 (8)	−0.0071 (8)	−0.0028 (8)
C22	0.0349 (12)	0.0397 (13)	0.0290 (11)	0.0059 (10)	−0.0154 (10)	−0.0088 (10)
C23	0.0332 (12)	0.0420 (13)	0.0376 (13)	0.0041 (10)	−0.0118 (10)	−0.0172 (11)
C24	0.0237 (10)	0.0283 (11)	0.0441 (13)	0.0027 (9)	−0.0071 (10)	−0.0092 (10)
C25	0.0307 (12)	0.0448 (14)	0.0380 (13)	0.0055 (10)	−0.0165 (10)	−0.0058 (11)
C26	0.0339 (12)	0.0408 (13)	0.0309 (12)	0.0046 (10)	−0.0143 (10)	−0.0093 (10)
C31	0.0396 (13)	0.0400 (13)	0.0322 (12)	−0.0071 (10)	−0.0117 (10)	0.0025 (10)
C32	0.0487 (15)	0.0456 (15)	0.0496 (16)	−0.0194 (12)	−0.0195 (13)	0.0046 (12)
C33	0.0574 (16)	0.0521 (16)	0.0462 (15)	−0.0155 (13)	−0.0264 (13)	−0.0077 (13)
C34	0.0454 (13)	0.0408 (13)	0.0294 (12)	−0.0044 (11)	−0.0171 (11)	−0.0063 (10)
C35	0.0262 (10)	0.0298 (11)	0.0256 (11)	0.0014 (8)	−0.0093 (9)	−0.0029 (9)
C36	0.0261 (10)	0.0311 (11)	0.0215 (10)	0.0036 (8)	−0.0075 (8)	−0.0021 (8)
C41	0.0225 (9)	0.0257 (10)	0.0219 (10)	0.0038 (8)	−0.0040 (8)	−0.0062 (8)
C42	0.0321 (11)	0.0331 (12)	0.0256 (11)	0.0001 (9)	−0.0097 (9)	−0.0018 (9)
C43	0.0335 (12)	0.0396 (13)	0.0335 (12)	−0.0007 (10)	−0.0151 (10)	−0.0064 (10)
C44	0.0252 (10)	0.0288 (11)	0.0378 (13)	−0.0027 (9)	−0.0055 (9)	−0.0071 (10)
C45	0.0339 (12)	0.0307 (12)	0.0319 (12)	0.0005 (9)	−0.0095 (10)	0.0021 (9)
C46	0.0281 (10)	0.0331 (11)	0.0259 (11)	0.0037 (9)	−0.0105 (9)	−0.0044 (9)
C51	0.0311 (11)	0.0295 (11)	0.0219 (10)	0.0025 (9)	−0.0103 (9)	−0.0048 (8)
C52	0.0357 (12)	0.0336 (12)	0.0265 (11)	−0.0013 (9)	−0.0116 (9)	−0.0035 (9)
C53	0.0325 (12)	0.0560 (16)	0.0278 (12)	−0.0060 (11)	−0.0088 (10)	−0.0011 (11)
C54	0.0312 (12)	0.0616 (17)	0.0320 (13)	0.0112 (11)	−0.0090 (10)	−0.0096 (12)
C55	0.0432 (13)	0.0406 (14)	0.0339 (13)	0.0133 (11)	−0.0100 (11)	−0.0091 (11)
C56	0.0336 (11)	0.0313 (12)	0.0299 (12)	0.0023 (9)	−0.0077 (9)	−0.0062 (9)
C61	0.0338 (11)	0.0226 (10)	0.0223 (10)	−0.0017 (8)	−0.0027 (9)	−0.0009 (8)
C62	0.0323 (11)	0.0276 (11)	0.0316 (12)	−0.0003 (9)	−0.0039 (9)	−0.0030 (9)
C63	0.0360 (12)	0.0308 (12)	0.0450 (14)	0.0070 (10)	−0.0024 (11)	−0.0048 (11)
C64	0.0508 (15)	0.0269 (12)	0.0446 (15)	0.0014 (11)	−0.0005 (12)	−0.0098 (11)
C65	0.0581 (16)	0.0299 (12)	0.0376 (13)	−0.0073 (11)	−0.0121 (12)	−0.0095 (10)
C66	0.0447 (13)	0.0274 (11)	0.0314 (12)	0.0002 (10)	−0.0116 (10)	−0.0035 (9)
C71	0.0301 (11)	0.0227 (10)	0.0298 (11)	−0.0008 (8)	−0.0137 (9)	−0.0062 (8)
C72	0.0379 (12)	0.0285 (11)	0.0297 (11)	0.0038 (9)	−0.0130 (10)	−0.0082 (9)
C73	0.0583 (16)	0.0289 (12)	0.0319 (12)	0.0040 (11)	−0.0194 (12)	−0.0018 (10)
C74	0.0543 (16)	0.0372 (14)	0.0307 (13)	−0.0051 (12)	−0.0073 (12)	0.0013 (10)
C75	0.0336 (12)	0.0465 (15)	0.0459 (15)	−0.0055 (11)	−0.0059 (11)	−0.0012 (12)
C76	0.0316 (12)	0.0373 (13)	0.0390 (13)	−0.0011 (10)	−0.0128 (10)	0.0014 (10)
C81	0.0370 (12)	0.0327 (12)	0.0350 (12)	0.0163 (9)	−0.0189 (10)	−0.0142 (10)
C82	0.0527 (15)	0.0594 (17)	0.0346 (13)	0.0339 (13)	−0.0205 (12)	−0.0190 (12)
C83	0.085 (2)	0.105 (3)	0.0425 (17)	0.067 (2)	−0.0395 (17)	−0.0407 (18)
C84	0.099 (3)	0.117 (3)	0.088 (3)	0.079 (3)	−0.074 (2)	−0.079 (3)
C85	0.079 (2)	0.068 (2)	0.124 (3)	0.0302 (18)	−0.072 (2)	−0.062 (2)
C86	0.0624 (17)	0.0432 (15)	0.074 (2)	0.0143 (13)	−0.0445 (16)	−0.0260 (14)
B	0.0315 (12)	0.0233 (11)	0.0259 (12)	0.0030 (9)	−0.0121 (10)	−0.0046 (9)

### Geometric parameters (Å, °)

**Table:**

Br1—C24	1.896 (2)	C45—C46	1.388 (3)
Br2—C44	1.897 (2)	C45—H45	0.9500
Cu—N1	2.0158 (17)	C46—H46	0.9500
Cu—N4	2.0237 (16)	C51—C56	1.402 (3)
Cu—N3	2.0278 (17)	C51—C52	1.405 (3)
Cu—N2	2.0331 (16)	C51—B	1.648 (3)
N1—C11	1.339 (3)	C52—C53	1.395 (3)
N1—C15	1.354 (3)	C52—H52	0.9500
N2—C16	1.281 (3)	C53—C54	1.379 (4)
N2—C21	1.425 (3)	C53—H53	0.9500
N3—C31	1.335 (3)	C54—C55	1.381 (3)
N3—C35	1.354 (3)	C54—H54	0.9500
N4—C36	1.285 (2)	C55—C56	1.387 (3)
N4—C41	1.424 (3)	C55—H55	0.9500
C11—C12	1.389 (3)	C56—H56	0.9500
C11—H11	0.9500	C61—C62	1.404 (3)
C12—C13	1.366 (3)	C61—C66	1.404 (3)
C12—H12	0.9500	C61—B	1.646 (3)
C13—C14	1.376 (3)	C62—C63	1.389 (3)
C13—H13	0.9500	C62—H62	0.9500
C14—C15	1.389 (3)	C63—C64	1.379 (4)
C14—H14	0.9500	C63—H63	0.9500
C15—C16	1.463 (3)	C64—C65	1.378 (4)
C16—H16	0.9500	C64—H64	0.9500
C21—C22	1.391 (3)	C65—C66	1.394 (3)
C21—C26	1.391 (3)	C65—H65	0.9500
C22—C23	1.384 (3)	C66—H66	0.9500
C22—H22	0.9500	C71—C72	1.395 (3)
C23—C24	1.379 (3)	C71—C76	1.411 (3)
C23—H23	0.9500	C71—B	1.652 (3)
C24—C25	1.382 (3)	C72—C73	1.401 (3)
C25—C26	1.378 (3)	C72—H72	0.9500
C25—H25	0.9500	C73—C74	1.379 (3)
C26—H26	0.9500	C73—H73	0.9500
C31—C32	1.382 (3)	C74—C75	1.378 (4)
C31—H31	0.9500	C74—H74	0.9500
C32—C33	1.375 (4)	C75—C76	1.383 (3)
C32—H32	0.9500	C75—H75	0.9500
C33—C34	1.381 (3)	C76—H76	0.9500
C33—H33	0.9500	C81—C86	1.387 (3)
C34—C35	1.380 (3)	C81—C82	1.406 (3)
C34—H34	0.9500	C81—B	1.647 (3)
C35—C36	1.463 (3)	C82—C83	1.380 (4)
C36—H36	0.9500	C82—H82	0.9500
C41—C42	1.388 (3)	C83—C84	1.362 (5)
C41—C46	1.390 (3)	C83—H83	0.9500
C42—C43	1.386 (3)	C84—C85	1.385 (5)
C42—H42	0.9500	C84—H84	0.9500
C43—C44	1.381 (3)	C85—C86	1.403 (4)
C43—H43	0.9500	C85—H85	0.9500
C44—C45	1.377 (3)	C86—H86	0.9500
			
N1—Cu—N4	135.35 (7)	C44—C45—C46	119.5 (2)
N1—Cu—N3	120.47 (7)	C44—C45—H45	120.2
N4—Cu—N3	81.51 (7)	C46—C45—H45	120.2
N1—Cu—N2	82.60 (7)	C45—C46—C41	119.99 (19)
N4—Cu—N2	122.20 (7)	C45—C46—H46	120.0
N3—Cu—N2	119.62 (7)	C41—C46—H46	120.0
C11—N1—C15	116.88 (18)	C56—C51—C52	114.76 (19)
C11—N1—Cu	131.53 (14)	C56—C51—B	118.81 (18)
C15—N1—Cu	111.15 (13)	C52—C51—B	126.31 (19)
C16—N2—C21	120.81 (17)	C53—C52—C51	122.4 (2)
C16—N2—Cu	111.51 (14)	C53—C52—H52	118.8
C21—N2—Cu	127.49 (13)	C51—C52—H52	118.8
C31—N3—C35	118.01 (18)	C54—C53—C52	120.5 (2)
C31—N3—Cu	129.64 (15)	C54—C53—H53	119.7
C35—N3—Cu	112.25 (13)	C52—C53—H53	119.7
C36—N4—C41	120.62 (17)	C53—C54—C55	118.9 (2)
C36—N4—Cu	112.89 (14)	C53—C54—H54	120.5
C41—N4—Cu	126.46 (13)	C55—C54—H54	120.5
N1—C11—C12	123.3 (2)	C54—C55—C56	120.0 (2)
N1—C11—H11	118.4	C54—C55—H55	120.0
C12—C11—H11	118.4	C56—C55—H55	120.0
C13—C12—C11	119.0 (2)	C55—C56—C51	123.4 (2)
C13—C12—H12	120.5	C55—C56—H56	118.3
C11—C12—H12	120.5	C51—C56—H56	118.3
C12—C13—C14	119.2 (2)	C62—C61—C66	114.67 (19)
C12—C13—H13	120.4	C62—C61—B	122.13 (18)
C14—C13—H13	120.4	C66—C61—B	122.54 (19)
C13—C14—C15	118.9 (2)	C63—C62—C61	122.9 (2)
C13—C14—H14	120.5	C63—C62—H62	118.5
C15—C14—H14	120.5	C61—C62—H62	118.5
N1—C15—C14	122.72 (19)	C64—C63—C62	120.3 (2)
N1—C15—C16	115.62 (17)	C64—C63—H63	119.9
C14—C15—C16	121.57 (19)	C62—C63—H63	119.9
N2—C16—C15	119.06 (18)	C65—C64—C63	119.1 (2)
N2—C16—H16	120.5	C65—C64—H64	120.4
C15—C16—H16	120.5	C63—C64—H64	120.4
C22—C21—C26	119.3 (2)	C64—C65—C66	120.0 (2)
C22—C21—N2	116.76 (18)	C64—C65—H65	120.0
C26—C21—N2	123.91 (18)	C66—C65—H65	120.0
C23—C22—C21	120.6 (2)	C65—C66—C61	122.9 (2)
C23—C22—H22	119.7	C65—C66—H66	118.5
C21—C22—H22	119.7	C61—C66—H66	118.5
C24—C23—C22	119.0 (2)	C72—C71—C76	114.5 (2)
C24—C23—H23	120.5	C72—C71—B	125.94 (19)
C22—C23—H23	120.5	C76—C71—B	119.46 (18)
C23—C24—C25	121.0 (2)	C71—C72—C73	122.9 (2)
C23—C24—Br1	119.73 (17)	C71—C72—H72	118.6
C25—C24—Br1	119.22 (17)	C73—C72—H72	118.6
C26—C25—C24	119.7 (2)	C74—C73—C72	120.2 (2)
C26—C25—H25	120.1	C74—C73—H73	119.9
C24—C25—H25	120.1	C72—C73—H73	119.9
C25—C26—C21	120.1 (2)	C75—C74—C73	118.8 (2)
C25—C26—H26	119.9	C75—C74—H74	120.6
C21—C26—H26	119.9	C73—C74—H74	120.6
N3—C31—C32	122.5 (2)	C74—C75—C76	120.4 (2)
N3—C31—H31	118.8	C74—C75—H75	119.8
C32—C31—H31	118.8	C76—C75—H75	119.8
C33—C32—C31	119.1 (2)	C75—C76—C71	123.2 (2)
C33—C32—H32	120.5	C75—C76—H76	118.4
C31—C32—H32	120.5	C71—C76—H76	118.4
C32—C33—C34	119.3 (2)	C86—C81—C82	116.0 (2)
C32—C33—H33	120.3	C86—C81—B	125.4 (2)
C34—C33—H33	120.3	C82—C81—B	118.6 (2)
C35—C34—C33	118.5 (2)	C83—C82—C81	122.3 (3)
C35—C34—H34	120.8	C83—C82—H82	118.9
C33—C34—H34	120.8	C81—C82—H82	118.9
N3—C35—C34	122.58 (19)	C84—C83—C82	120.8 (3)
N3—C35—C36	114.60 (17)	C84—C83—H83	119.6
C34—C35—C36	122.83 (19)	C82—C83—H83	119.6
N4—C36—C35	118.61 (18)	C83—C84—C85	119.0 (3)
N4—C36—H36	120.7	C83—C84—H84	120.5
C35—C36—H36	120.7	C85—C84—H84	120.5
C42—C41—C46	119.52 (19)	C84—C85—C86	120.2 (3)
C42—C41—N4	117.29 (18)	C84—C85—H85	119.9
C46—C41—N4	123.18 (17)	C86—C85—H85	119.9
C43—C42—C41	120.7 (2)	C81—C86—C85	121.7 (3)
C43—C42—H42	119.7	C81—C86—H86	119.2
C41—C42—H42	119.7	C85—C86—H86	119.2
C44—C43—C42	118.91 (19)	C61—B—C81	110.40 (17)
C44—C43—H43	120.5	C61—B—C51	114.88 (17)
C42—C43—H43	120.5	C81—B—C51	104.67 (16)
C45—C44—C43	121.4 (2)	C61—B—C71	104.24 (16)
C45—C44—Br2	118.99 (17)	C81—B—C71	111.07 (17)
C43—C44—Br2	119.61 (16)	C51—B—C71	111.72 (16)
			
N4—Cu—N1—C11	56.5 (2)	Cu—N4—C41—C42	29.4 (2)
N3—Cu—N1—C11	−54.1 (2)	C36—N4—C41—C46	32.7 (3)
N2—Cu—N1—C11	−174.2 (2)	Cu—N4—C41—C46	−149.60 (16)
N4—Cu—N1—C15	−131.45 (13)	C46—C41—C42—C43	0.0 (3)
N3—Cu—N1—C15	117.95 (14)	N4—C41—C42—C43	−178.97 (19)
N2—Cu—N1—C15	−2.18 (13)	C41—C42—C43—C44	1.1 (3)
N1—Cu—N2—C16	1.75 (14)	C42—C43—C44—C45	−1.2 (3)
N4—Cu—N2—C16	141.75 (14)	C42—C43—C44—Br2	176.76 (17)
N3—Cu—N2—C16	−119.20 (14)	C43—C44—C45—C46	0.2 (3)
N1—Cu—N2—C21	176.71 (17)	Br2—C44—C45—C46	−177.81 (16)
N4—Cu—N2—C21	−43.30 (18)	C44—C45—C46—C41	1.0 (3)
N3—Cu—N2—C21	55.75 (18)	C42—C41—C46—C45	−1.1 (3)
N1—Cu—N3—C31	−41.1 (2)	N4—C41—C46—C45	177.86 (19)
N4—Cu—N3—C31	−179.4 (2)	C56—C51—C52—C53	1.7 (3)
N2—Cu—N3—C31	58.3 (2)	B—C51—C52—C53	177.76 (19)
N1—Cu—N3—C35	135.06 (14)	C51—C52—C53—C54	−1.0 (3)
N4—Cu—N3—C35	−3.25 (14)	C52—C53—C54—C55	−0.6 (3)
N2—Cu—N3—C35	−125.58 (14)	C53—C54—C55—C56	1.3 (3)
N1—Cu—N4—C36	−123.84 (14)	C54—C55—C56—C51	−0.5 (3)
N3—Cu—N4—C36	1.50 (14)	C52—C51—C56—C55	−1.0 (3)
N2—Cu—N4—C36	121.28 (14)	B—C51—C56—C55	−177.4 (2)
N1—Cu—N4—C41	58.28 (18)	C66—C61—C62—C63	1.3 (3)
N3—Cu—N4—C41	−176.38 (16)	B—C61—C62—C63	−169.6 (2)
N2—Cu—N4—C41	−56.60 (17)	C61—C62—C63—C64	−1.1 (4)
C15—N1—C11—C12	−0.9 (3)	C62—C63—C64—C65	0.1 (4)
Cu—N1—C11—C12	170.75 (17)	C63—C64—C65—C66	0.6 (4)
N1—C11—C12—C13	1.8 (4)	C64—C65—C66—C61	−0.3 (4)
C11—C12—C13—C14	−0.8 (4)	C62—C61—C66—C65	−0.6 (3)
C12—C13—C14—C15	−1.0 (4)	B—C61—C66—C65	170.3 (2)
C11—N1—C15—C14	−1.0 (3)	C76—C71—C72—C73	1.6 (3)
Cu—N1—C15—C14	−174.33 (17)	B—C71—C72—C73	−174.54 (19)
C11—N1—C15—C16	175.56 (18)	C71—C72—C73—C74	−0.6 (3)
Cu—N1—C15—C16	2.3 (2)	C72—C73—C74—C75	−0.5 (4)
C13—C14—C15—N1	2.0 (3)	C73—C74—C75—C76	0.6 (4)
C13—C14—C15—C16	−174.4 (2)	C74—C75—C76—C71	0.5 (4)
C21—N2—C16—C15	−176.37 (17)	C72—C71—C76—C75	−1.5 (3)
Cu—N2—C16—C15	−1.0 (2)	B—C71—C76—C75	174.9 (2)
N1—C15—C16—N2	−0.9 (3)	C86—C81—C82—C83	0.2 (3)
C14—C15—C16—N2	175.8 (2)	B—C81—C82—C83	−177.6 (2)
C16—N2—C21—C22	158.5 (2)	C81—C82—C83—C84	0.2 (4)
Cu—N2—C21—C22	−16.1 (3)	C82—C83—C84—C85	−0.2 (4)
C16—N2—C21—C26	−20.7 (3)	C83—C84—C85—C86	−0.1 (4)
Cu—N2—C21—C26	164.72 (16)	C82—C81—C86—C85	−0.6 (3)
C26—C21—C22—C23	2.5 (3)	B—C81—C86—C85	177.1 (2)
N2—C21—C22—C23	−176.7 (2)	C84—C85—C86—C81	0.6 (4)
C21—C22—C23—C24	1.0 (3)	C62—C61—B—C81	−25.8 (3)
C22—C23—C24—C25	−3.4 (4)	C66—C61—B—C81	163.98 (19)
C22—C23—C24—Br1	176.13 (17)	C62—C61—B—C51	−143.88 (19)
C23—C24—C25—C26	2.3 (4)	C66—C61—B—C51	45.9 (3)
Br1—C24—C25—C26	−177.25 (17)	C62—C61—B—C71	93.5 (2)
C24—C25—C26—C21	1.3 (3)	C66—C61—B—C71	−76.7 (2)
C22—C21—C26—C25	−3.7 (3)	C86—C81—B—C61	124.4 (2)
N2—C21—C26—C25	175.5 (2)	C82—C81—B—C61	−58.0 (2)
C35—N3—C31—C32	−1.7 (3)	C86—C81—B—C51	−111.4 (2)
Cu—N3—C31—C32	174.20 (19)	C82—C81—B—C51	66.2 (2)
N3—C31—C32—C33	0.9 (4)	C86—C81—B—C71	9.3 (3)
C31—C32—C33—C34	0.7 (4)	C82—C81—B—C71	−173.12 (18)
C32—C33—C34—C35	−1.4 (4)	C56—C51—B—C61	−176.98 (18)
C31—N3—C35—C34	0.9 (3)	C52—C51—B—C61	7.1 (3)
Cu—N3—C35—C34	−175.68 (17)	C56—C51—B—C81	61.8 (2)
C31—N3—C35—C36	−179.05 (19)	C52—C51—B—C81	−114.1 (2)
Cu—N3—C35—C36	4.3 (2)	C56—C51—B—C71	−58.5 (2)
C33—C34—C35—N3	0.6 (3)	C52—C51—B—C71	125.6 (2)
C33—C34—C35—C36	−179.4 (2)	C72—C71—B—C61	121.5 (2)
C41—N4—C36—C35	178.46 (17)	C76—C71—B—C61	−54.4 (2)
Cu—N4—C36—C35	0.4 (2)	C72—C71—B—C81	−119.6 (2)
N3—C35—C36—N4	−3.3 (3)	C76—C71—B—C81	64.5 (2)
C34—C35—C36—N4	176.7 (2)	C72—C71—B—C51	−3.1 (3)
C36—N4—C41—C42	−148.37 (19)	C76—C71—B—C51	−179.04 (18)
